# Missed opportunities: Public health messaging in media coverage of drug seizures

**DOI:** 10.1016/j.puhip.2025.100601

**Published:** 2025-03-14

**Authors:** Sunyou Kang, Jule von der Heydt, Renuka Anjali Joshi-Dave, Julia Papasodoro, Leo Beletsky, Bradley Ray

**Affiliations:** a The Action Lab, School of Law and Bouvé College of Health Sciences, Northeastern University, Boston, MA, United States; b RTI International, 3040 Cornwallis Road, Research Triangle Park, NC, United States

## Abstract

**Objective:**

To examine mainstream media coverage of drug seizures and identify trends in messaging on substance use treatment and other public health responses.

**Study design:**

We compiled news reports published January 2022–May 2024 on drug seizures in the United States.

**Methods:**

We extracted information on incident trends (including geography, drugs and other items seized, agencies involved, and mentions of substance use treatment-related resources).

**Results:**

Only three of 211 articles (1 %) had any mention of substance use treatment or other public health-related resources. Of those three articles, only one provided actionable information linking to resources.

**Conclusions:**

Drug seizure-related media coverage is a missed opportunity to prevent drug-related harms. The lack of public health messaging in drug seizure-related media coverage should be rectified by refocusing coverage away from drug enforcement narratives and instead provide guidance towards evidence-based resources and services.

## 1 Introduction

**Fig. 1:**
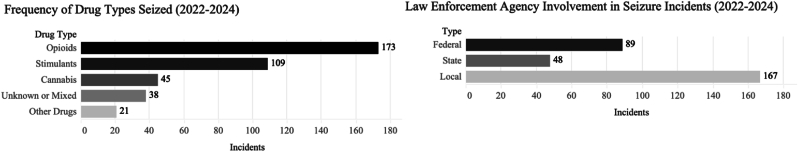
Drug types seized and agency involvement by number of incidents (2022–2024). NOTES: Information was extracted from a sample of 211 news articles. “Incidents” refers to reports of drug seizure. “Other drugs” includes those not classified as opioids, stimulants, cannabis, or substances of unknown or mixed composition.

A growing number of studies suggest unintended negative health consequences from law enforcement engagement with people who use drugs [1]. This includes research on periods of incarceration increasing the risk of overdose upon re-entry to community [2] but also increased overdose resulting from police confiscation of illicit substances, colloquially referred to as “drug seizures.” [3,4] A recent study detailed the “drug bust paradox,” [5] showing that drug seizures actually increased overdose rates in the surrounding community; in fact, fatal overdoses more than doubled within 7 days and 100 m of an opioid-related drug seizure [6]. This research supports others showing that people procuring illicit drugs in an unregulated market, especially from new suppliers, face uncertainties around content or potency and thus, increased risk of overdose [7] (see Fig. 1).

Decades of drug interdiction have resulted in market pressure to increase drug potency [7] but have not brought reductions in the price or availability of drugs. With evidence that enforcement contributes to drug-related harms, the National Drug Control Strategy has increased investment in a public health approach to the ongoing overdose epidemic by expanding access to treatment and recovery support services for substance use disorders [8]. A lack of reliable information on appropriate and evidence-based approaches to recovery contributes to the gaps in access to such resources. Authoritative sources of information such as mainstream media outlets and law enforcement have the potential to help close these gaps. However, the extent to which these figures advance credible drug-related information and resources is yet unknown.

Law enforcement has typically had a symbiotic relationship with mainstream media, amplifying police narratives as news, and sometimes at the expense of critical scrutiny. Moreover, drug-related media is rife with misinformation and notably lacking in perspectives of those with lived experience with substance use as well as accurate information on evidence-based treatment or recovery resources [9]. As such, we sought to characterize news media coverage of drug seizures and identify the extent to which public health-based narratives, such as information on substance use treatment and other recovery supports, were included.

## 2 Methods

Media Cloud, a web-scraping tool hosted by the MIT Media Lab, was used to compile news content published between January 2022 and May 2024 related to drug seizures. The following two Boolean queries were used: (drug∗ OR substance∗ OR “cannabis” OR “marijuana” OR opioid∗ OR “heroin” OR “fentanyl” OR “methamphetamine” OR “meth” OR “cocaine” or “pills”) AND (police OR “DEA” OR sheriff∗ OR “border patrol” OR “authorities” OR arrest∗); (“drug seizure”) or (“drug raid”) or (“drug bust”). We queried Media Cloud's National news collection, which hosts 249 of the most prominent U.S.-focused news sources. Four coders eliminated duplicate and irrelevant items, and from the resulting final sample, extracted incident date; incident location(s); additional incidents mentioned; drug types (opioids, stimulants, cannabis, or other) and quantity seized (in kg); arrests made; weapons seized; financial assets seized; mentions of substance use disorder treatment or recovery resources; and law enforcement agency involvement.

The information extracted pertains to the primary incident noted as the article's focus. Articles not mentioning a specific drug seizure were excluded (such as those on drug policy or statistics on drug trafficking). Articles mentioning multiple incidents as part of the same enforcement operation were coded as one entry with the first available incident date noted. For articles referring to multiple discrete, unrelated incidents, information on the primary incident of focus was coded and additional incidents mentioned were noted. Mentions of treatment were specifically coded based on the original article pulled by Media Cloud to identify narratives around treatment and recovery support in the national news landscape. The final sample was redundantly coded for accuracy, resulting in 211 relevant news articles (Online Data Supplement).

## 3 Results

Opioids were most frequently reported seized in our sample, confiscated in 173 incidents (45 % of total drug types seized), followed by stimulants (n = 109, 28 %), cannabis (n = 45, 12 %), substances of unknown or mixed composition (n = 38, 10 %), then “other” drug types (n = 21, 5 %) including benzodiazepines, psilocybin and other hallucinogens, medications for opioid use disorder, and steroids. Stimulants were most reported seized by weight, accounting for 55 % (4868 kg) of a total 8824 kg seized. Substances of unknown or mixed composition accounted for 23 % (2007 kg), followed by opioids (1562 kg, 18 %), cannabis (346 kg, 4 %), and other drug types (41 kg, 0.5 %).

Incidents and published articles peaked in the second half of 2022 and were noted across 34 states, with CA (n = 42, 20 %), NY (n = 24, 11 %), and CT (n = 18, 9 %) most represented (Appendix A). We calculated 1281 individual reported arrests across all incidents. Weapons were reported seized in 101 incidents (48 %), and cash or other financial assets such as electronic benefit transfer cards [10] in 76 incidents (36 %). Local law enforcement were involved in 167 incidents (79 %), federal law enforcement in 89 incidents (42 %), and state law enforcement in 48 incidents (23 %). Multi-level agency cooperation was reported in 70 incidents (33 %).

Nearly all stories followed a typical drug law enforcement perspective, framing seizures as positive public safety efforts to deter drug use through interdiction. Only three (1 %) articles had any mention of substance use treatment, recovery support services, or overdose prevention, including harm reduction resources. Those mentions were brief and only one offered actionable information for readers, providing contact information for a “substance abuse helpline” (Appendix A). One article noted partnership between the sheriff's department and the county's department of behavioral health, and the other referred to a portion of pharmaceutical settlement funds to be used for treatment.

## 4 Discussion

With only three (1 %) of this media sample mentioning resources or guidance post-seizure, there is a clear opportunity and need to shift public health messaging to center the unintended and largely unrecognized harms of drug seizures. Currently, the coverage of drug seizures is used to suggest effectiveness of enforcement and reinforce support for punitive measures which are at direct cross-purposes with evidence-based public health interventions [9]. For example, headlines referring to millions of lives saved by a single seizure are proliferating through the media landscape and were present in our sample [9].

Misinformation and fear-invoking language are hallmarks of drug policy coverage [9]. As one example, myths around the risks of touching or inhaling fentanyl linger in the media landscape – and though fentanyl-related misinformation was not a focus of this study, our sample was no exception. Additionally, images of confiscated drugs, weapons, and cash captioned with emphasis on potential lives saved are there to engender support for increased drug enforcement. With only three articles providing information on engaging with treatment or other recovery support services (Appendix A), there is an opportunity to refocus drug policy and enforcement-related discourse toward providing links to meaningful resources to address overdose.

## 5 Public health implications

With the recognition that extant work has established the unintended harms of interdiction on overdose outcomes, it is concerning to have found a notable lack of public health messaging in drug seizure-related media coverage. Systematic efforts should be made across sectors to redirect attention away from fear-mongering narratives, taking precedence from historical examples of large-scale fact-checking (such as with COVID-19-related misinformation). As just one example, augmenting publishing standards and providing trainings for journalists in ethical and evidence-based media coverage can recalibrate the media landscape with evidence-based information. Providing guardrails for media coverage of particularly sensitive topics is critical for directing attention and resources towards post-seizure guidance that includes substance use treatment and recovery support.

## Author statements

There were no human subjects in this study, and it was determined exempt from IRB. This study was supported by a research grant from the CDC (R01CE003362) to Dr. Ray. None of the authors have any competing interests to report and all authors contributed to the manuscript through idea generation, data collection and coding, and reviewing and editing all drafts of the manuscript.

## Funding

We would like to thank the 10.13039/100000030Centers for Disease Control and Prevention as part of grant number R01CE003362.

## Declaration of competing nterest

The authors have no conflicts of interest to report at this time.

## Appendix A

Article excerpts mentioning substance use treatment or recovery resources:

“VCSD has partnered with Ventura county Behavioral Health to reduce overdoses and educate residents about the dangers of opioids and other drugs.”^1^

“For help: Call the Lines for Life substance abuse helpline at 1-800-923-4357 or go to www.linesforlife.org. Phone support is available 24 h a day, seven days a week. You can also text “RecoveryNow” to 839863 between 8 a.m. and 11 p.m. daily.”^2^

“Bonta said the state was not interested in ‘restarting the war on drugs,’ by putting those struggling with addiction behind bars but rather getting them treatment. In addition to the busts, settlements between the state Department of Justice and pharmaceutical companies have netted $32 billion nationwide, with $2 billion specifically for California, ‘bringing much needed funding back to communities for treatment and prevention strategies,’ Bonta said.”^3^
